# Predicting Working Memory in Healthy Older Adults Using Real-Life Language and Social Context Information: A Machine Learning Approach

**DOI:** 10.2196/28333

**Published:** 2022-03-08

**Authors:** Andrea Ferrario, Minxia Luo, Angelina J Polsinelli, Suzanne A Moseley, Matthias R Mehl, Kristina Yordanova, Mike Martin, Burcu Demiray

**Affiliations:** 1 Chair of Technology Marketing ETH Zurich Zurich Switzerland; 2 Mobiliar Lab for Analytics ETH Zurich Zurich Switzerland; 3 Department of Psychology University of Zurich Zurich Switzerland; 4 University Research Priority Program Dynamics of Healthy Aging University of Zurich Zurich Switzerland; 5 Department of Neurology Indiana University School of Medicine Indianapolis, IN United States; 6 Department of Psychology Minnesota Epilepsy Group St Paul, MN United States; 7 Department of Psychology University of Arizona Tucson, AZ United States; 8 Institute of Computer Science University of Rostock Rostock Germany

**Keywords:** cognitive aging, language complexity, social context, machine learning, natural language processing, Electronically Activated Recorder (EAR), behavioral indicators

## Abstract

**Background:**

Language use and social interactions have demonstrated a close relationship with cognitive measures. It is important to improve the understanding of language use and behavioral indicators from social context to study the early prediction of cognitive decline among healthy populations of older adults.

**Objective:**

This study aimed at predicting an important cognitive ability, working memory, of 98 healthy older adults participating in a 4-day-long naturalistic observation study. We used linguistic measures, part-of-speech (POS) tags, and social context information extracted from 7450 real-life audio recordings of their everyday conversations.

**Methods:**

The methods in this study comprise (1) the generation of linguistic measures, representing idea density, vocabulary richness, and grammatical complexity, as well as POS tags with natural language processing (NLP) from the transcripts of real-life conversations and (2) the training of machine learning models to predict working memory using linguistic measures, POS tags, and social context information. We measured working memory using (1) the Keep Track test, (2) the Consonant Updating test, and (3) a composite score based on the Keep Track and Consonant Updating tests. We trained machine learning models using random forest, extreme gradient boosting, and light gradient boosting machine algorithms, implementing repeated cross-validation with different numbers of folds and repeats and recursive feature elimination to avoid overfitting.

**Results:**

For all three prediction routines, models comprising linguistic measures, POS tags, and social context information improved the baseline performance on the validation folds. The best model for the Keep Track prediction routine comprised linguistic measures, POS tags, and social context variables. The best models for prediction of the Consonant Updating score and the composite working memory score comprised POS tags only.

**Conclusions:**

The results suggest that machine learning and NLP may support the prediction of working memory using, in particular, linguistic measures and social context information extracted from the everyday conversations of healthy older adults. Our findings may support the design of an early warning system to be used in longitudinal studies that collects cognitive ability scores and records real-life conversations unobtrusively. This system may support the timely detection of early cognitive decline. In particular, the use of a privacy-sensitive passive monitoring technology would allow for the design of a program of interventions to enable strategies and treatments to decrease or avoid early cognitive decline.

## Introduction

### Cognitive Ability, Its Decline, and Older Adults’ Behaviors

Cognitive abilities play a crucial role in the daily functioning of older adults [1]. Although decline in certain cognitive abilities is expected in the course of normal aging, some individuals may go on to experience decline to an extent that is pathological, namely mild cognitive impairment (MCI) or dementia [2,3]. It is argued that subtle changes in older adults’ everyday behaviors may occur in the preclinical stage [4]. As such, behavioral indicators may provide an important avenue for detecting cognitive decline in this population. Some studies have started to quantify differences in the everyday activities (eg, medication intake and telephone use) of older adults experiencing normal aging versus those in pathological aging by observing participants and using manual reporting [5,6].

These methods could aid in detecting behavioral changes; however, they are also prone to human error, including recall bias [7]. Thus, the approach of visiting a health care professional for an examination may end up preventing older adults and their caregivers from continuously monitoring and proactively reacting to cognitive decline [8]. In fact, older adults visit health care professionals to receive cognitive examinations, such as cognitive assessment tests, blood tests, and structural imaging [9]. However, this may happen when the cognitive decline has become severe enough to disrupt daily functioning. In these cases, it is often too late for them to receive effective treatments and to make preventive plans with their families [10,11].

### The Use of Technology to Predict Early Cognitive Decline in Real Life

To detect cognitive decline at an early stage, some recent studies have considered using technology to collect behavioral data from real-life settings, focusing on cognitively healthy older adults and those who have MCI [8,12].

For example, comparing the behaviors of healthy older adults with those with MCI, Seelye et al [13] collected 1 week of computer mouse movements. Their results showed that older adults with MCI had fewer total mouse moves and longer pauses between movements. In another study, Seelye et al [14] examined driving behaviors observed from a driving sensor and showed that older adults with MCI drove fewer miles and spent less time on the highway per day than those without MCI. To try understanding behavioral variability in normal aging, Austin et al [15] focused on word use in the internet searches of healthy older adults in a 6-month-long study with home-based unobtrusive technology. Their results showed that older adults with higher cognitive abilities used more unique words than older adults with lower cognitive abilities. Therefore, they argued that collecting the terms people use in internet searches may aid in detection of early cognitive decline [15].

The use of technology to collect objective behavioral indicators in real-life settings shows a few advantages with respect to clinical settings. It allows for generating high-frequency data over extended periods of time, offering more data than the assessments performed during appointments with health care professionals. High-frequency data could provide an objective baseline to understand individuals’ own norms of behaviors that could be used to detect early cognitive decline [16]. Moreover, collecting behavioral indicators in real-life settings by means of technology empowers older adults and caregivers to monitor and detect cognitive decline, freeing them from the exclusive reliance on examinations by health care professionals. It could also help patients and caregivers to predict early changes in cognitive abilities. This could help reduce stress in caregivers, allowing them to better manage time and perform advanced planning [10]. Low-cost and unobtrusive technology methods have the potential to be applied to large-scale community studies for identifying at-risk populations [17]. However, to leverage the advantages offered by technology in the early detection of cognitive decline it is necessary to identify reliable behavioral indicators of cognitive decline for different populations of older adults (ie, healthy older adults and those with MCI or dementia) that can be effectively and unobtrusively monitored over time.

### Linguistic Measures as Behavioral Indicators of Cognitive Decline

Linguistic measures elicited from speech are one type of behavioral indicator that have proved to be useful in predicting cognitive abilities. To this end, studies have considered the use of linguistic measures from transcribed speeches of healthy subjects, or those with different degrees of cognitive impairment in structured clinical assessments [18]. In fact, it has been shown that language markers predict normal and pathological cognitive functioning [19]. Typically, these studies are conducted in the lab, with elicitation of speech through clinical interviews and the recording of cognitive function scores via batteries of validated tests. For example, Fraser et al [20] examined various linguistic features, such as part-of-speech (POS) tags, grammatical complexity, vocabulary richness, and repetitiveness, and showed them to be useful in predicting dementia cases. Furthermore, more and more studies have focused on differences in language use between healthy older adults and those with MCI [19,21], with the aim of facilitating the detection of cognitive decline at an early stage [22].

Although linguistic markers captured from lab-based speech samples have shown promise in detecting cognitive decline, the limitations of these speech samples must be considered. For example, studying language in clinical settings through its elicitation may result in the generation of utterances that are not representative of daily language use. This may lead to a biased understanding of the cognitive abilities of the aging population [23]. Moreover, in clinical settings it is not possible to study the participants’ social contexts. These contexts offer opportunities for older adults to engage in cognitively stimulating activities and they are protective of their cognitive abilities [24-26].Therefore, we argue that research focusing on the early decline of cognitive abilities would benefit from (1) considering everyday life settings where cognitive abilities are expressed and (2) collecting everyday language use and information on the social contexts of healthy older adults by means of unobtrusive monitoring technology.

As a first step in this direction, Polsinelli et al [27] recently tested whether healthy older adults’ language in their everyday lives provides information about cognitive processes. In their study, Polsinelli et al assessed the cognitive abilities of healthy older adults with a battery of tests, including the testing of working memory. Working memory refers to the cognitive ability of maintaining input information while simultaneously performing complex tasks with this information, such as reasoning, communication, and learning [28]. It is an important aspect of fluid intelligence for the production of complex language [29].

They sampled real-life ambient audio data from participants’ naturally occurring daily lives, transcribed the conversations captured in the ambient audio sound bites, and applied natural language processing (NLP); in their case, they used Linguistic Inquiry and Word Count [30], a very widely used and extensively validated closed vocabulary–based text analysis approach. With respect to protecting the privacy of participants and their bystanders, they followed a set of established procedures that included providing participants an opportunity to censor (ie, delete) selected recordings and alerting conversation partners about the possibility of their conversations being recorded, thereby ensuring passive consent [31,32]. Their results show that higher working memory was associated “with analytic, complex, and specific language” [27].

On the other hand, in examining age effects in language use using verbatim transcripts derived from real-life ambient audio recordings, Luo and colleagues [33,34] recently showed that healthy older adults produced more complex language with familiar conversational partners (eg, spouse, friends, and family) than with strangers, and more complex language in substantive conversations than in small talk. These findings support the assumption that some social contexts offer opportunities for cognitively stimulating activities. Thus, healthy older adults’ social contexts may provide useful information for predicting their cognitive abilities over time.

### Using Machine Learning and NLP to Predict Healthy Older Adults’ Working Memory

Polsinelli et al’s [27] and Luo et al’s [33,34] studies suggest that the language use and social contexts encoded in everyday life ambient audio data may support the understanding of healthy older adults’ cognitive abilities. This is seen as a first step toward an improved understanding of cognitive decline by means of information collected in everyday life. Therefore, in this paper we explore the possibility of predicting cognitive ability, namely working memory, by combining linguistic measures, including POS tags, and social context information computed from the verbatim transcripts of the sampled everyday conversations of healthy older adults using machine learning and NLP. In this study, the term “healthy older adults” is meant as “cognitively healthy older adults.” The conversations were transcribed from the real-life ambient audio data that were recorded unobtrusively using a smartphone app [35]. We consider the data from Polsinelli et al’s original study [27], where working memory was measured using two separate tests, namely Keep Track and Consonant Updating [27,36]. Therefore, in this study, we predicted working memory using Keep Track, Consonant Updating, and a combined score (ie, the mean score from Keep Track and Consonant Updating) [27,36]. To the best of our knowledge, this is the first study where machine learning and NLP are used to predict selected cognitive abilities of healthy older adults combining different sources of information, such as linguistic measures and social context, extracted from data collected in a naturalistic observation setting.

In future studies, the methods described in this paper could support the design of passive monitoring systems to detect early cognitive decline by recording, ultimately in a privacy-sensitive way (ie, protecting the content and context of the actual “raw” conversations), real-life ambient audio data and using information extracted from the everyday conversations of older adults. Systems with reliable performance may allow for designing intervention programs aimed at coping with early signs of cognitive decline in normal aging as well as at the preclinical stage of Alzheimer disease. This technology and intervention programs would, therefore, empower older adults and caregivers to monitor and detect cognitive decline autonomously. Low-cost and unobtrusive technologies have the potential to be applied to large-scale community studies for identifying at-risk populations [17]. This is in line with the recommendations of the World Health Organization’s 2020 report on the global action of “Decade of Healthy Ageing 2020-2030,” which states that technologies can empower older people to monitor and understand their own health, enabling greater decision-making about their own lives by tracking their trajectories of healthy aging [37].

## Methods

### Data Collection

Data used in this study originated within Moseley’s [36] and Polsinelli’s [38] dissertations and were studied by Polsinelli et al [27]. All participants from the original studies were community-dwelling individuals recruited from the greater Tucson, Arizona, community in the United States. Participants were recruited via community events and via research databases from prior and ongoing studies in the Department of Psychology and the Department of Speech, Language, and Hearing Sciences at the University of Arizona. Participants’ living situations included retirement communities; mobile home communities; single-family homes, with and without a live-in partner; and residences in family members’ homes, usually children.

All participants were cognitively healthy older adults, with no reported history of neurologic or psychiatric disorders. Polsinelli et al’s [27] sample consisted of 102 participants (mean age 75.8 years, SD 5.8; mean years of education 16.5, SD 2.3; 54.9% [n=56] female; 62.7% [n=64] married). During the study, participants underwent cognitive testing in the lab and wore the Electronically Activated Recorder (EAR) app [31,35] that was installed on provided smartphones for 4.5 days of their daily lives. The EAR enables frequent, passive, and unobtrusive sampling of participants’ language use in their natural environments via ambient recording [35,39,40]. The EAR was set to record 30-second audio files every 12 minutes (ie, five times per hour), except for a 6-hour overnight period. At the end of the study, after returning the EAR, all participants completed a standard EAR evaluation measure [27,39]. Polsinelli et al [27] collected 31,683 valid (ie, adherent and codable) and waking (ie, nonsleeping) sound files.

Recording raw ambient sounds raises important questions around privacy. Polsinelli et al’s study implemented several safeguards to protect the privacy of participants and conversation partners. First, the audio sampling limited the net recording to a small fraction of the day (<5%), keeping the vast majority of conversations private in the first place. Second, the short recordings (ie, 30 seconds) ensured that minimal personal information was captured beyond what was necessary for reliable coding. Third, participants could review their recordings and censor (ie, delete) any they wished to remain private. Fourth, a “warning triangle” was placed visibly on the recording device to alert conversation partners of the possibility of being recorded, in order to ensure passive consent. Finally, the study was covered by a National Institutes of Health Certificate of Confidentiality, which protects the data against forced third-party disclosure. In implementing these procedures, the study followed the established guidelines for passive ambient audio sampling [31,32].

### Data Generation: Measuring Working Memory

In this study, we considered working memory as measured by the Keep Track and Consonant Updating tests [36,38]. These are select subtests from Miyake et al [41] that served as the guiding model of working memory and executive functioning more broadly. During the Keep Track test [27,41,42], participants view a list of 15 serially presented words, that is, presented one at a time (eg, banana, golf, uncle, and so on). They are instructed to hold in mind the last word that is presented in predefined categories (eg, fruits, sports, and relatives). Initially, participants keep track of one category, but over duration of the test, they increase to keeping track of four categories, with three trials for each number of categories (eg, three trials of one category, three trials of two categories, and so on) [27]. Participants write down the last word they remembered from each predefined category, before moving on to the next trial.

In the Consonant Updating test [27,36,41], participants are required to say aloud the last four letters in a string of consonants appearing on the screen [27]. Each trial in the Consonant Updating test consists of five, seven, nine, or 11 letters in random order, for a total of 108 participant responses. In Polsinelli et al’s study [27], 4 participants only completed the Keep Track test; in this work, we include only the 98 participants who completed both tests.

### Data Generation: Transcribing and Coding Audio Files

In Polsinelli et al’s study [27], a team of research assistants were trained to listen to each 30-second audio file, identify the participant’s voice, and transcribe verbatim the spoken utterances only of the participants (ie, they did not transcribe speech from nonparticipants). Out of 31,683 audio files, 7450 contained snippets of conversations. Concurrently, research assistants coded for multiple behavioral and contextual variables. Codes were binary, indicating either presence (“1”) or absence (“0”) of a variable within the entire 30-second audio file. While audio files were coded for multiple variables, only the 19 variables relevant to this investigation are described here. These 19 variables, called “social context variables” in what follows, fall into the following overarching categories: environment (ie, in public or on the phone), presence or absence of social partners (ie, alone, with one person, or with multiple people), conversation partner (ie, self, pet, significant other, close friend or family member, acquaintance, or stranger), conversation type (ie, small talk, substantive conversation, or gossip), and activity (ie, socializing or entertaining, watching TV, eating or drinking, doing housework, or in transit).

For more detailed information on how EAR sound files are coded for daily behavior, we refer to Kaplan et al’s work [43].

### NLP of Transcripts: Linguistic Measures and Part-of-Speech Tags

In this study, we included three domains of linguistic measures that have been commonly examined in the cognitive aging literature. The first domain is idea density, also known as proposition density, representing the number of ideas that are expressed [44]. Studies show that idea density declines over age in both normal and pathological aging [44]. We computed idea density with the CPIDR (Computerized Propositional Idea Density Rater) software (version 5) [45]. The second domain is vocabulary richness, indicating usage of unique words. In this study, it was represented by the measure of entropy with the Chao-Shen estimator [46]. We computed vocabulary richness using the “entropy” package from R (The R Foundation) [47]. The third domain is grammatical complexity, indicating how complex the grammatical structures are [34,44]. We computed the scores with the syntactic complexity analyzer [48,49] in R. We focused on the measures of clauses and dependent clauses (ie, number of clauses, number of dependent clauses, mean length clause, and dependent clause ratio).

In this study, the measures computed from the aforementioned domains of linguistic measures are referred to as “linguistic measures.” In addition to the linguistic measures, we also considered POS tags of written transcripts. POS tagging is the procedure that assigns a POS tag to each word in a corpus of textual data [50,51]. The POS tag encodes information on the role of the word and its context. In this study, we used the spaCy library in Python (Python Software Foundation) [52] to retrieve the POS tags for each word in all of the 7450 transcripts. The data set comprises 15 distinct POS tags.

### Machine Learning

#### Feature Generation and Data Aggregation

To perform machine learning modeling and predict individual working memory scores, we aggregated the data set of 7450 transcripts at the participant level, arriving at 98 data points. We proceeded with the aggregation of the features as follows. Sociodemographic features (ie, age, sex, marital status, and education) were not aggregated, as they are constant for each participant. Linguistic measures were aggregated by computing the mean and SD of the distribution of the language measures of all transcripts for each participant. In addition, we concatenated the POS tags extracted from all transcripts of each participant. Finally, social context features (eg, “alone”) were aggregated by computing the percentage of transcripts in which the social context was detected (eg, “alone = 1”) for each participant. We collected all features resulting from data aggregation in Multimedia Appendix 1.

#### Target Variables

In this study, we aimed at gathering a foundational understanding of the problem of predicting working memory with information extracted from real-life audio data. Therefore, we considered three distinct machine learning regression problems. First, we predicted the standard scores of the Keep Track test for each participant. Second, we predicted the standard scores of the Consonant Updating test for each participant. Finally, we standardized the mean score of the Keep Track and Consonant Updating tests for each participant. This latter score measured working memory for each participant. The use of standard scores (ie, z scores) for cognitive ability tests is in line with previous studies in the literature [15,27]. However, we remark that we computed standard scores inside the repeated cross-validation routine on each training fold (see Experimental Setting section) to avoid “data leakage,” as recommended by Hastie et al [53].

#### Machine Learning Models

We considered random forest (RF), extreme gradient boosting (XGBoost), and light gradient boosting machine (LightGBM) algorithms [54-57] for this study, using their Python implementations. We chose them due to the possibility to consider different hyperparameter combinations and to explain results using feature importance scores. The RF feature importance score computes the mean (across all trees in the forest) Gini impurity decrease for the feature at hand: the higher the decrease, the higher the feature importance. The XGBoost and LightGBM feature importance scores compute the number of times (in percentages) each feature is used to split the data across all trees of the ensemble. Moreover, different authors considered RF and XGBoost algorithms for the detection of reminiscence from transcripts of conversations of older adults [58,59]. Similarly, Yordanova et al [60] used RF algorithms to detect social behavior from transcripts of daily conversations.

#### Experimental Setting

##### Overview

We provide information on the experimental setting by describing the (1) machine learning runs (R, when reported with run number), (2) repeated cross-validation routine, (3) recursive feature elimination (RFE) algorithm, (4) hyperparameters in the cross-validation, and (5) the evaluation metrics of the machine learning models.

##### Machine Learning Runs

We considered eight different runs of machine learning modeling, each corresponding to a different combination of features. We present them in Table 1, together with the total number of features per run. R0 was considered the baseline for all machine learning runs, as it contained only sociodemographic variables (ie, age, education, marital status, and sex; Multimedia Appendix 1). We also note that sociodemographic variables were considered in all runs of this study as control variables.

**Table 1:** All runs considered in this study.

Run	Feature combination	Features, n
R0	Sociodemographic	4
R1	Sociodemographic + linguistic measures	18
R2	Sociodemographic + social context	23
R3	Sociodemographic + POS^a^ tags	19
R4	Sociodemographic + linguistic measures + social context	37
R5	Sociodemographic + social context + POS tags	38
R6	Sociodemographic + linguistic measures + POS tags	33
R7	Sociodemographic + linguistic measures + social context + POS tags	52

^a^POS: part of speech.

As our study dealt with a limited number of data points (ie, n=98), machine learning modeling needed to avoid the use of too many noisy variables and incur overfitting. This would lower reproducibility of results and their applicability to unseen data [61]. Moreover, in the presence of a small number of data points, resampling techniques, such as cross-validation, may show high variance. Therefore, we needed to introduce a routine to select the best-performing machine learning model by doing the following: (1) using resampling techniques such as cross-validation, (2) reducing the variance of cross-validation, and (3) performing feature selection on all runs to prevent overfitting.

##### Repeated Cross-validation

Standard k-fold cross-validation divides a data set into k nonoverlapping subsets. Each model is trained on k–1 folds and evaluated on the k-th fold, for a total of k models. Model performance (eg, the mean squared error; see Evaluation Metrics section) is the mean of the performance on all k folds used for the evaluation. With a fixed training data set, k-fold cross-validation depends on the randomness of partitioning the training data set into k-folds [62]. This variance is also called internal variance [63,64]. In particular, in the context of small data sets, Braga-Neto and Dougherty [64] stated that cross-validation error estimation shows high variance, with the effect of making “individual estimates unreliable for small samples.”

Repeated k-fold cross-validation is a procedure introduced to reduce the internal variance of k-fold cross-validation routines. The procedure called “repeated k-fold cross-validation with n-repeats” simply repeats k-fold cross-validation N times, with different splits, and averages the model performances across all folds from all runs. It provides a performance evaluation of the model that is more robust than the one computed from a single run of k-fold cross-validation. It has been suggested due to its performance, but at the price of a steep computational cost [65]. We refer to the work by Krstajic et al [66], in particular Algorithm 1, for more details on repeated cross-validation.

Our strategy is to apply repeated cross-validation with 2, 5, and 10 folds, and a number of repeats equal to 50, 20, and 10, respectively. For each k, the number of repeats, N, is chosen to have a total of 2 × 50 = 5 × 20 = 10 × 10 = 100 validation folds for the evaluation of model performance. These fold values have been considered by Molinaro et al [61] in their comparison of resampling methods. A small number of folds increases the bias of the cross-validation estimator, but it is computationally efficient [67]. A higher number of folds decreases the bias but increases the variance, as the validation sets become smaller.

##### Recursive Feature Elimination

**Figure 1:**
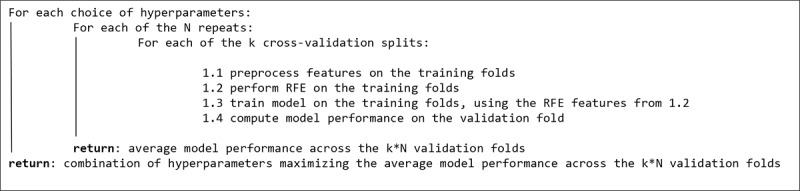
Repeated cross-validation with the recursive feature elimination (RFE) algorithm.

To avoid overfitting, we performed feature selection by implementing the RFE algorithm [68] embedded in the repeated cross-validation routine. We used it for all runs to select the machine learning model with the best performance on the 100 validation folds, choosing different numbers of features to select. We summarize the algorithm performing repeated cross-validation with RFE in Figure 1.

##### Hyperparameters in the Repeated Cross-validation

Table 2 summarizes all the hyperparameters tuned in the algorithm in Figure 1.

In particular, we preprocessed POS tags with term frequency–inverse document frequency (TF-IDF) normalization to use them as features in the machine learning modeling routines. We performed no hyperparameter tuning, by considering only 1-grams. The number of hyperparameter combinations depends on the machine learning run. For example, the best RF model for the R0 run emerged from fitting 4500 models. On the other hand, to select the best RF model for the R7 run, we fitted 220,000 models, following the algorithm in Figure 1. We then fit the model corresponding to the combination of hyperparameters from Figure 1 to the whole data set, following Algorithm 1 in Krstajic et al [66].

**Table 2:** Summary of all hyperparameters tuned in the repeated cross-validation with the RFE algorithm.

Algorithm or model	Hyperparameters
RFE^a^ algorithm	Number of features to select Number of features to reduce at each step
Machine learning model (RF^b^)	Number of trees Maximum tree depth
Machine learning model (XGBoost^c^ and LightGBM^d^)	Number of trees Maximum tree depth Learning rate

^a^RFE: recursive feature elimination.

^b^RF: random forest.

^c^XGBoost: extreme gradient boosting.

^d^LightGBM: light gradient boosting machine.

##### Evaluation Metrics

The performance of each model in the repeated cross-validation with the RFE algorithm in Figure 1 was evaluated by computing the mean and SD of the distribution of the mean squared errors (MSEs) on each of the 100 validation folds. The MSE was computed as follows (*q* denotes the number of data points in the validation fold):




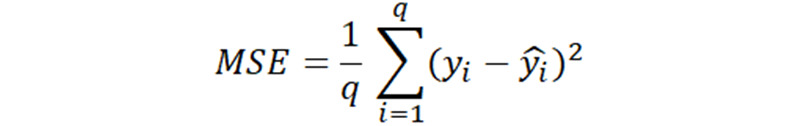




We used the MSE as the scoring method for the cross-validation. As we implemented the standardization of scores inside the repeated cross-validation routine, it follows that the MSE on the validation folds was computed using unstandardized scores.

### Ethics Consideration

Participants whose data were studied by Polsinelli et al [27] gave permission for their data to be used in future research studies (Institutional Review Board No. 1300000709).

## Results

### Predicting Keep Track

In Table 3, we present the best models resulting from the repeated cross-validation with the RFE algorithm in Figure 1 for the Keep Track target variable. All results are obtained for 10 folds and 10 repeats. By definition of Polsinelli et al’s experimental setting [27], the total number of recalled words during the test was 30. In this study, the mean of the Keep Track scores in the data set was 19 (SD 3.6); the minimum and maximum Keep Track scores were 10 and 27.

**Table 3:** Performance of the best models for the prediction of the Keep Track target variable. All results were obtained for 10 folds and 10 repeats.

Run	Model	MSE^a^, mean (SD)	Features, n
R0	LightGBM^b^	13.26 (5.33)	4
R1	LightGBM	12.80 (5.43)	10
R2	LightGBM	12.46 (4.85)	5
R3	LightGBM	12.95 (4.98)	10
R4^c^	LightGBM	11.81 (4.92)	10
R5	LightGBM	12.12 (4.43)	20
R6	LightGBM	12.65 (4.92)	15
R7	LightGBM	12.02 (4.66)	25

^a^MSE: mean squared error.

^b^LightGBM: light gradient boosting machine.

^c^The best run was R4.

All runs improved performance with respect to the baseline (ie, R0). The best run was R4, which delivered an improvement of 11% in mean MSE on the validation folds with respect to R0. The resulting LightGBM model was an ensemble of 70 trees, with a maximum depth equal to 1. Moreover, the RFE algorithm selected 10 features for this model out of 37 (27%), as per Table 1, deleting 50% of features at each step. As seen at the end of the Results section, the model improved the mean MSE by 13% on the validation folds with respect to the constant model that predicted the Keep Track scores on the validation fold using the mean on the training fold, for each of the 100 splits.

Table 4 shows all of the 8 features out of 10 (80%) in the best LightGBM model for R4 with nonzero importance and their type. All three feature types (ie, sociodemographic, linguistic measure, and social context) were represented in the model. More than half of the features were of the social context type. The mean feature importance was 0.13. The most important features were the percentage of transcripts for which each participant was alone (ie, “alone_prc”), the age of the participant (ie, “age at EAR testing”), the mean of the distribution of the idea density of the transcripts per participant (ie, “mean_Density”), and the SD of the distribution of Chao-Shen–corrected entropies of transcript per participant (ie, “std_ChaoShen”).

**Table 4:** Features, their importance, and type for the best light gradient boosting machine model of R4 for prediction of Keep Track scores.

Rank	Feature^a^	Importance of feature	Type of feature
1	alone_prc	0.34	Social context
2	age at EAR^b^ testing	0.16	Sociodemographic
3	mean_Density	0.13	Linguistic measure
4	std_ChaoShen	0.13	Linguistic measure
5	TV_prc	0.10	Social context
6	in_transit_prc	0.07	Social context
7	partner_sign_other_prc	0.04	Social context
8	small_talk_prc	0.03	social context

^a^Descriptions of features are listed in Multimedia Appendix 1.

^b^EAR: Electronically Activated Recorder.

### Predicting Consonant Updating

In Table 5, we present the best models resulting from the repeated cross-validation with the RFE algorithm in Figure 1 for the Consonant Updating prediction task. As opposed to the best RF models in Table 3, in the case of Consonant Updating, the best models in different runs were obtained in the presence of different k values of cross-validation folds.

The mean Consonant Updating score in the data set was 24 (SD 10.6), and the minimum and maximum Consonant Updating scores were 0 and 45, respectively.

All runs, with the exception of R1, R2, and R4, improved performance with respect to the baseline (ie, R0). The best run was R3, where the LightGBM model delivered an improvement of 14% in mean MSE on the validation folds with respect to R0. The LightGBM model was an ensemble of 30 shallow trees with a depth equal to 1. The RFE algorithm selected only 5 out of the 19 (26%) available features for R3 (Table 1), deleting 10% of the features at each step.

**Table 5:** Performance of the best models for the prediction of the Consonant Updating target variable.

Run	k	Model	MSE^a^, mean (SD)	Features, n
R0	10	LightGBM^b^	113.50 (45.55)	4
R1	5	LightGBM	114.85 (25.64)	18
R2	5	LightGBM	114.00 (26.04)	5
R3^c^	5	LightGBM	97.26 (21.38)	5
R4	10	LightGBM	114.30 (45.50)	10
R5	5	LightGBM	100.73 (22.93)	5
R6	5	LightGBM	100.07 (22.74)	5
R7	10	XGBoost^d^	101.38 (41.32)	5

^a^MSE: mean squared error.

^b^LightGBM: light gradient boosting machine.

^c^The best run was R3.

^d^XGBoost: extreme gradient boosting.

As seen at the end of the Results section, the best model improved the mean MSE by 15% on the validation folds with respect to the constant model that predicted the Consonant Updating scores on the validation fold using the mean of the scores on the training fold, for each of the 100 splits. Table 6 shows the nonzero feature importance for R3 of the LightGBM model (ie, the best model). All features were POS tags, namely “NUM” (ie, numeral), “INTJ” (ie, interjection), “NOUN,” (ie, noun), and “ADP” (ie, adposition).

**Table 6:** Features, their importance, and type for the best light gradient boosting machine model of R3 for prediction of Consonant Updating scores.

Rank	Feature	Importance of feature	Type of feature
1	NUM	0.37	Part of speech
2	INTJ	0.23	Part of speech
3	NOUN	0.23	Part of speech
4	ADP	0.17	Part of speech

### Predicting Working Memory

In Table 7, we present the best models resulting from the repeated cross-validation with the RFE algorithm in Figure 1 for the prediction task of Working Memory. As in the case of Consonant Updating, the best models in different runs were obtained in the presence of different k values of cross-validation folds.

Similar to the prediction of the Consonant Updating scores, all runs, with the exception of R2 and R4, improved performance with respect to the baseline (ie, R0). The best run was R3, where the best XGBoost model delivered an improvement of 20% in mean MSE on the validation folds with respect to R0. The XGBoost model was an ensemble of 30 trees with a depth equal to 1. The RFE algorithm selected only 10 out of the 19 (53%) available features for R3 (Table 1), deleting 50% of the features at each step. The R5 and R6 best models showed almost equal performance and the same number of features.

As seen at the end of the Results section, the best model improved the mean MSE by 20% on the validation folds with respect to the constant model that predicted the Working Memory scores on the validation fold using the mean scores on the training fold, for each of the 100 splits. In Table 8, we show the 6 features with nonzero feature importance; they are the same as those for the best model predicting Consonant Updating, with the addition of the “PRON” (ie, pronoun) and “PROPN” (ie, proper noun) POS tags. In Table 9, the best models from Tables 3, 5, and 7 are benchmarked with the constant model predicting the mean value of the target variable for all three predictions.

**Table 7:** Performance of the best models for the prediction of the Working Memory target variable.

Run	k	Model	MSE^a^, mean (SD)	Features, n
R0	5	LightGBM^b^	37.75 (7.94)	4
R1	10	LightGBM	37.70 (14.07)	10
R2	5	LightGBM	37.75 (7.93)	5
R3^c^	5	XGBoost^d^	30.23 (6.63)	10
R4	5	LightGBM	37.75 (7.93)	5
R5	10	XGBoost	31.49 (13.03)	5
R6	10	LightGBM	31.25 (12.24)	5
R7	5	XGBoost	32.22 (6.77)	5

^a^MSE: mean squared error.

^b^LightGBM: light gradient boosting machine.

^c^The best run was R3.

^d^XGBoost: extreme gradient boosting.

**Table 8:** Features, their importance, and type for the best extreme gradient boosting model of R3 for the prediction of Working Memory scores.

Rank	Feature	Importance of feature	Type of feature
1	NUM	0.30	Part of speech
2	INTJ	0.20	Part of speech
3	NOUN	0.20	Part of speech
4	PRON	0.13	Part of speech
5	ADP	0.10	Part of speech
6	PROPN	0.07	Part of speech

**Table 9:** Benchmarking the best models from Tables 3, 5, and 7 with the constant model predicting the mean value of the target variable for all three predictions.

Prediction	MSE^a^ of constant model, mean (SD)	MSE of best model, mean (SD)
Keep Track	13.57 (5.37)	11.81 (4.92)
Consonant Updating	114.77 (45.71)	97.26 (21.38)
Working Memory	37.81 (14.05)	30.23 (6.63)

^a^MSE: mean squared error.

## Discussion

### Summary of the Prediction Tasks

We applied machine learning methodologies to Polsinelli et al’s study [27] to predict cognitive ability, namely working memory, by means of the scores on the Keep Track and Consonant Updating tasks and a composite of both (ie, Working Memory). The best model for the Keep Track prediction exercise comprised sociodemographic, linguistic measure, and social context variables. Those for Consonant Updating and Working Memory comprised POS tags only. Our methodologies delivered an improvement of performance with respect to two baseline models (ie, the models using only sociodemographic variables and the models predicting the mean value of the target variable) for all three prediction tasks. All of the best models were gradient boosting ensembles: LightGBM for Keep Track and Consonant Updating, and XGBoost for Working Memory. All ensembles comprised “tree stumps” (ie, trees with only one split), and they made use of a limited number of features.

### Feature Analysis for All Prediction Tasks

Considering the prediction of Keep Track scores, the high importance of social context variables in the model was in line with previous studies on the effects of social context on cognitive aging. Specifically, Luo [34] reported that older adults produce more complex language with their significant others than with strangers. Familiarity with significant others may have enabled more diverse conversation topics than talking with strangers. More diverse conversation topics may have offered more opportunities to engage in cognitively stimulating conversations and, thus, protect against cognitive decline. By contrast, a higher occurrence of nonsocial contexts, such as watching TV and being alone, indicated deprived opportunities for engaging in cognitively stimulating activities. Fancourt and Steptoe’s [69] study showed that watching TV for more than 3.5 hours per day is related to cognitive decline in older adults. Moreover, social isolation has been shown to be associated with memory decline in old age [70]. The best model for predicting Keep Track scores indicated that the corresponding social context variables are important, in an ensemble of regression trees, in machine learning problems aimed at predicting working memory.

We note that “mean_Density” was the only linguistic measure with high feature importance, together with the SD of the distribution of the Chao-Shen–corrected entropies of transcript per participant (ie, “std_ChaoShen”). This finding is in line with previous literature, where idea density has been commonly used to predict cognitive decline in older adults [18,22].

Considering sociodemographic variables, only the age of the participants (ie, “age at EAR testing”) was retrieved by the RFE algorithm for the best model in the Keep Track prediction. It showed a feature importance (ie, 0.16) that was higher than the mean of the distribution. We note that age was a significant variable in the models by Austin et al [15]. Interestingly, neither the sex, the marital status, nor the number of years of education of each of the participants appeared as features in the best models for all three prediction tasks. This is a point of difference with respect to Austin et al’s results [15].

Finally, POS tags—via the generation of bag-of-words features using TF-IDF normalization—featured prominently in the prediction of Consonant Updating and Working Memory. This finding may suggest that how older adults structure their sentences (eg, encoded in the use of prepositions, which expresses relations between different concepts [27]) in their daily conversations reveals the integrity of aspects of their working memory. This is different than the prediction of Keep Track scores, where features, such as the counts of different social contexts coded from the transcripts, were also predictive. In particular, in both of the best models for Consonant Updating and Working Memory, the most important POS tag was “NUM” (ie, “numerals”). The POS tags “INTJ,” “NOUN,” “PRON,” “ADP,” and “PROPN” (ie, “interjection,” “noun,” “pronoun,” “adposition,” and “proper noun,” respectively) also appeared in the models. We argue that their presence may indicate that recorded conversations showed a certain degree of variability, as recently detected in studies with the EAR device [25]. We also note that, in particular, interjections (eg, “oh,” “uh,” “yeah,” and “uhm”) are commonly used in the spoken language to shift the attention to the speaker or as a back-channel response in conversations.

The original Polsinelli et al study [27] also found that selected POS tags correlated with working memory, using a partial Spearman correlation analysis. Some of these POS tags were also important predictors in this study, including numbers, which featured prominently in two of our three models, and prepositions. In particular, in the case of numerals, the authors found statistically significant Spearman partial correlation (*r*=0.32, range 0.13-0.48) between working memory measures and the use of numbers in everyday conversations [27]. The replication is encouraging and warrants further investigation. As highlighted in the original Polsinelli et al study, prepositions are a component of more complex language, and it is possible that this complexity is associated with working memory. However, at this time, without clear theoretical reasons for the predictive power of specific POS tags, we are cautious about overspeculating and overinterpreting these data. It will be important for future work to replicate these findings in an unrelated sample to assist in better understanding these POS markers of working memory. It may be especially interesting to examine the broader context in which certain POS are used; for example, numbers may be used in the context of someone paying bills or doing taxes, which are behaviors likely associated with aspects of cognition, including working memory.

Results from this study provide preliminary evidence to support the prediction of an important cognitive ability, working memory, by (1) collecting behavior from everyday conversations of healthy older adults in a naturalistic setting using the EAR app, (2) generating different families of behavioral features, and (3) using machine learning methodologies, with automated feature selection routines and combining families of behavioral features. In particular, the machine learning methodologies went beyond the correlations between working memory and POS tags from Polsinelli et al’s study [27] and showed how different sets of features generated from the transcripts of conversations predict cognition. The approach in this study can be used in everyday settings to collect linguistic measures and social context information using unobtrusive technology.

Using this methodology, it may be possible to design an early warning system for cognitive decline in older adults that uses samples of conversations in daily life. In fact, one of the largest challenges in the current cognitive aging field is early detection for early intervention. This methodology may be one potential tool for addressing this problem through early and continuous monitoring over months or even years.

Continuous monitoring could result in near-immediate notification—to the individual, to the individual’s family, or to a health care provider—when there is a suggestion of decline. In this way, an individual would be identified much earlier on in the process of potential decline and could seek a full professional evaluation in a much timelier manner, thereby increasing access to care and intervention. It is also possible that these “alerts” from continuous monitoring could reduce help-seeking delays caused by fear or anxiety of diagnosis [10]. The results could supplement a comprehensive clinical assessment, offering reliable and ecologically valid objective information to support formal diagnosis [12]. The continuous collection of high-frequency data could also serve as useful baseline information for clinicians to understand the rate of cognitive decline or to determine effectiveness of treatments [16].

However, we highlight that older adults and their caregivers may express concern about threats that are potentially posed by sensing technologies and opaque machine learning methodologies in digital health, such as threats on autonomy, privacy, and freedom [71] and their effects on the trustworthiness of these systems [71,72]. Yet, research has shown that it is possible to gain understanding from the users when they are provided with sufficient knowledge about technologies and the possibility of knowledgeable participations [73]. In particular, the EAR method has established protocols to inform participants about study procedures and to enable participants to review their own recordings, providing ethical safeguard measures and a low level of obtrusiveness [31]. The EAR method has been used to collect data from older adults, and they rated the method with a low level of obtrusiveness [39]. Taken together, we argue that the EAR method, in combination with machine learning techniques, could be developed as a promising tool for monitoring and detecting cognitive change in older age.

### Comparison With Previous Work

Previous research has investigated the relationship between natural speech, language, and cognitive functions in the context of preclinical Alzheimer disease, or other forms of dementia, by means of speech, NLP, and machine learning. The literature abounds in examples of different speech and language measures that intercept different phonetic, syntactic, and semantic aspects of natural speech to predict for different levels of MCI with machine learning classifiers. However, these studies are typically conducted in clinical settings [19,73,74]. While assessment in a clinical setting has clear benefits (eg, increased control and standardization), it is limited in its ability to capture the full ecology of a person’s rich social life, including behaviors, language, and interactions in different social contexts and with different social partners.

On the other hand, naturalistic observation studies and the use of passive, mobile monitoring technology may assist in capturing “reliable contextual observations, made in more ecologically valid environments than purely the consulting room” [75] and generate high volumes of data. Polsinelli et al [27] have examined the “association between spontaneous, conversational language use in daily life and higher-order cognitive functioning in older adults without known cognitive impairment.” In particular, they found that working memory “was associated with analytic (e.g., more articles and prepositions), complex (e.g., more longer words), and specific (e.g., more numbers) language” [27]. Therefore, one may argue that changes in language (ie, increasing use of more general words such as “thing” instead of a specific object name) could be potential behavioral markers of cognitive decline. Should an individual or his family members observe such changes in language or other changes in cognition (ie, memory decline), this may be the impetus for discussion with a doctor who may decide to refer them for a formal neuropsychological evaluation to determine the presence of cognitive impairment.

In the vein of naturalistic observation, but not interpersonal interactions, others have sought to use at-home technology device usage to monitor cognitive performance in older adults. Austin et al [15] investigated the relationship between internet searches and cognitive ability in older adults in a cross-sectional study. They continuously monitored the terms that 42 cognitively healthy older adults entered in internet search engines over a 6-month period by means of “an unobtrusive home-based assessment platform” [15]. The authors reported a total of 2915 searches and a median of 22 searches per participant over the 6-month period [15]. Their study showed the applicability of continuous unobtrusive home-based monitoring technology to possibly detect cognitive decline in older adults. In fact, their results showed that higher cognitive ability scores were associated with more unique search terms entered per search and that higher cognitive abilities were associated with the use of more obscure words, as measured with word obscurity, during searches [15]. To compare the behaviors of older adults with and without MCI, Lyons et al [8] examined computer mouse movements and showed that older adults with MCI had fewer total mouse moves and longer pauses between movements. Moreover, Seelye et al [14] examined driving behaviors observed from a driving sensor and showed that older adults with MCI drove fewer miles and spent less time on the highway per day than those without MCI. Finally, Piau et al [17] conducted a literature review of digital biomarker technologies for MCI or early-stage Alzheimer disease detection in home-based settings. Their review showed that technology using embedded passive sensors may support research on early decline of cognitive abilities among large populations.

The use of naturalistic settings allows for the planning of longitudinal studies to detect early symptoms of cognitive decline using machine learning and unobtrusive technology. However, we note that coding is a resource-intensive process, in terms of both the time and cost of human labor, that necessitates trained resources to generate high-quality codes. It becomes infeasible in the presence of high volumes of data. An alternative explored by Yordanova et al [60] is to automate the coding of social behaviors from the transcripts of everyday conversations using machine learning and NLP. However, a fully automated analysis of recorded conversations of older adults would also necessitate of a system to automatically detect speech and generate transcriptions that may also incur errors.

### Limitations

This study has several limitations. The data set of transcripts had a limited number of records, as the naturalistic observation study [27] comprised 4 days of data collection and only 98 participants. We argue that the limited sample size affected the variability of contexts that were encoded in the transcripts and, ultimately, the performance of the machine learning models.

This said, we implemented a single cross-validation protocol for model selection and assessment due to the high number of runs, algorithms, and prediction exercises under consideration. However, this procedure may incur bias in reporting performance results [66]. Therefore, in future studies, we will consider using procedures, such as repeated stratified nested cross-validation [66], together with RFE to improve reporting of model performance.

Moreover, our work was based on a single naturalistic observation study. Therefore, future studies are planned to investigate the generalizability of its results.

Additionally, we did not aim at detecting *changes* in cognitive ability, as Polsinelli et al [27] performed cognitive ability tests once, for all participants. In this study, we focused on computing different families of features and combining them in multiple runs of machine learning modeling. Therefore, we considered three algorithms only (ie, RF, XGBoost, and LightGBM) to predict working memory. In future studies, we plan to use more advanced models (eg, neural networks) and to collect higher volumes of data. Finally, as in Polsinelli et al [27], we computed the cognitive ability of working memory using Keep Track and Consonant Updating scores, as well as their composite, called Working Memory. Therefore, in future studies we will consider predicting scores of other tests [76] and focus on other aspects of executive functioning [41].

### Conclusions

Results from this study support the use of linguistic measure and social context information from the transcripts of everyday conversations to predict cognitive ability, namely working memory, in healthy older adults. Several studies have assessed the relationship between cognitive abilities and linguistic measures. However this research is somewhat limited by data collection in clinical interview settings. Alternatively, the approach in this study allows us to use everyday settings to collect and process linguistic measures and social context information using unobtrusive technology. This provides preliminary evidence for the design and deployment of early warning systems that use everyday samples of conversations to predict cognitive decline in older adults. The detection of early cognitive decline may allow for the design of intervention programs to assist older adults, their families, and the health care system in coping with cognitive decline.

## Appendix

Multimedia Appendix 1 Descriptions of all study variables.

## Abbreviations

CPIDR: Computerized Propositional Idea Density Rater
EAR: Electronically Activated Recorder
LightGBM: light gradient boosting machine
MCI: mild cognitive impairment
MSE: mean squared error
NLP: natural language processing
POS: part of speech
R: run (when reported with run number)
RF: random forest
RFE: recursive feature elimination
TF-IDF: term frequency–inverse document frequency
XGBoost: extreme gradient boosting
