# Supplementary material for: Predicting Working Memory in Healthy Older Adults Using Real-Life Language and Social Context Information: A Machine Learning Approach
Source: JMIR Aging. 2022 Mar 8;5(1):e28333. doi: 10.2196/28333 (PMC8941438; doi:10.2196/28333)
Supplement: Multimedia Appendix 1 [file aging_v5i1e28333_app1.docx]

**Multimedia Appendix 1**

We collect all the variables used in the study.

**Table S1: Socio-demographic variables**

| **Variable** | **Description** |
| --- | --- |
| age at EAR testing | Age |
| sex_M | = 1 if male, 0 otherwise |
| married?_1 | = 1 if married, 0 otherwise |
| education | Number of years of education |

**Table S2: Linguistic measures**

| **Variable** | **Description** |
| --- | --- |
| mean_Density | Mean idea density of all of a participant’s transcripts |
| std_Density | Standard deviation of the idea density of all of a participant’s transcripts |
| mean_W | Mean number of words of all of a participant’s transcripts |
| std_W | Standard deviation of the number of words of all of a participant’s transcripts |
| mean_C | Mean number of clauses of all of a participant’s transcripts |
| std_C | Standard deviation of the number of clauses of all of a participant’s transcripts |
| mean_DC | Mean number of dependent clauses of all of a participant’s transcripts |
| std_DC | Standard deviation of the number of dependent clause of all of a participant’s transcripts |
| mean_MLC | Mean of the mean length clause of all of a participant’s transcripts |
| std_MLC | Standard deviation of the mean length clause of all of a participant’s transcripts |
| mean_DC.C | Mean number of the dependent clause ratio of all of a participant’s transcripts |
| std_DC.C | Standard deviation of the dependent clause ratio of all of a participant’s transcripts |
| mean_ChaoShen | Mean Chao-Shen entropy estimator of all of a participant’s transcripts |
| std_ChaoShen | Standard deviation of the Chao-Shen entropy estimator of all of a participant’s transcripts |

**Table S3: Social context variables**

| **Variable** | **Description** |
| --- | --- |
| alone | =1 if the participant is alone, 0 otherwise |
| w/ one person | =1 if the participant is with another person, 0 otherwise |
| w/ multiple people | =1 if the participant is with multiple people, 0 otherwise |
| phone | =1 if the participant is talking at the phone, 0 otherwise |
| in public | =1 if the participant is in public, 0 otherwise |
| self | =1 if the participant is talking with him/herself, 0 otherwise |
| partner/Sig. Other | =1 if the participant is talking with the partner or the significant other, 0 otherwise |
| unspecifed close other | =1 if the participant is talking with an unspecified close other person (e.g., brother, daughter), 0 otherwise |
| aquaintance | =1 if the participant is talking with an acquaintance, 0 otherwise |
| stranger | =1 if the participant is talking with a stranger, 0 otherwise |
| pet | =1 if the participant is talking to a pet, 0 otherwise |
| socializing/entertaining | =1 if the participant is engaged in a socializing or entertaining conversation, 0 otherwise |
| housework | =1 if the participant is engaged in housework, 0 otherwise |
| small talk | =1 if the participant is having a small talk, 0 otherwise |
| substantive convo | =1 if the participant is having a substantive conversation, 0 otherwise |
| gossip | =1 if the participant is gossiping, 0 otherwise |
| in transit | =1 if the participant is in transit, 0 otherwise |
| eat/drink | =1 if the participant is eating and/or drinking, 0 otherwise |
| TV | =1 if the participant is watching TV, 0 otherwise |

**Table S4: Aggregated social context variables**

| **Variable** | **Description** |
| --- | --- |
| alone_prc | The percentage of transcripts for which the social context “alone” returns “alone=1,” per each participant |
| w/ one person_prc | The percentage of transcripts for which the social context variable “w/ one person” returns “w/ one person=1,” per each participant |
| w/ multiple people_prc | The percentage of transcripts for which the social context variable “w/ multiple people” returns “w/ multiple people=1,” per each participant |
| phone_prc | The percentage of transcripts for which the social context variable “phone” returns “phone=1,” per each participant |
| in public_prc | The percentage of transcripts for which the social context variable “In public” returns “In public=1,” per each participant |
| self_prc | The percentage of transcripts for which the social context variable “Self” returns “Self=1,” per each participant |
| partner/sig. other_prc | The percentage of transcripts for which the social context variable “Partner/Sig. Other” returns “Partner/Sig. Other=1,” per each participant |
| unspecifed close other_prc | The percentage of transcripts for which the social context variable “Unspecifed close other” returns “Unspecifed close other=1,” per each participant |
| aquaintance_prc | The percentage of transcripts for which the social context variable “Aquaintance” returns “Aquaintance =1,” per each participant |
| stranger_prc | The percentage of transcripts for which the social context variable “Stranger” returns “Stranger =1,” per each participant |
| pet_prc | The percentage of transcripts for which the social context variable “Pet” returns “Pet=1,” per each participant |
| socializing/entertaining_prc | The percentage of transcripts for which the social context variable “Socializing/ Entertaining” returns “Socializing/ Entertaining =1,” per each participant |
| housework_prc | The percentage of transcripts for which the social context variable “Housework” returns “Housework=1,” per each participant |
| small talk_prc | The percentage of transcripts for which the social context variable “Small Talk” returns “Small Talk=1,” per each participant |
| substantive convo_prc | The percentage of transcripts for which the social context variable “Substantive Convo” returns “Substantive Convo =1,” per each participant |
| gossip_prc | The percentage of transcripts for which the social context variable “Gossip” returns “Gossip=1,” per each participant |
| in transit_prc | The percentage of transcripts for which the social context variable “In transit” returns “In transit=1,” per each participant |
| eat/drink_prc | The percentage of transcripts for which the social context variable “Eat/ Drink” returns “Eat/ Drink=1,” per each participant |
| TV_prc | The percentage of transcripts for which the social context variable “TV” returns “TV=1,” per each participant |

**Table S5: POS-tags**

| **Variable** | **Description** |
| --- | --- |
| INTJ | Interjection |
| PUNCT | Punctuation |
| VERB | Verb |
| PART | Particle |
| NOUN | Noun |
| ADP | Adposition |
| DET | Determiner |
| ADJ | Adjective |
| PRON | Pronoun |
| ADV | Adverb |
| NUM | Numeral |
| AUX | Auxiliary |
| CCONJ | Coordinating conjunction |
| PROPN | Proper noun |
| INTJ | Interjection |
| SCONJ | Subordinating conjunction |
| SYM | Symbol |
